# Virtual Point Control for Step-Down Perturbations and Downhill Slopes in Bipedal Running

**DOI:** 10.3389/fbioe.2020.586534

**Published:** 2020-12-18

**Authors:** Özge Drama, Alexander Badri-Spröwitz

**Affiliations:** Dynamic Locomotion Group, Max Planck Institute for Intelligent Systems, Stuttgart, Germany

**Keywords:** bipedal locomotion, postural stability (postural control), virtual point control, step-down perturbation, downhill running

## Abstract

Bipedal running is a difficult task to realize in robots, since the trunk is underactuated and control is limited by intermittent ground contacts. Stabilizing the trunk becomes even more challenging if the terrain is uneven and causes perturbations. One bio-inspired method to achieve postural stability is the virtual point (VP) control, which is able to generate natural motion. However, so far it has only been studied for level running. In this work, we investigate whether the VP control method can accommodate single step-down perturbations and downhill terrains. We provide guidelines on the model and controller parameterizations for handling varying terrain conditions. Next, we show that the VP method is able to stabilize single step-down perturbations up to 40 cm, and downhill grades up to 20–40° corresponding to running speeds of 2–5 ms^−1^. Our results show that the VP approach leads to asymmetrically bounded ground reaction forces for downhill running, unlike the commonly-used symmetric friction cone constraints. Overall, VP control is a promising candidate for terrain-adaptive running control of bipedal robots.

## 1. Introduction

Generating dynamic motion for biped robots is challenging due to the hybrid and non-linear dynamics of legged locomotion. The literature presents two main approaches to motion planning: the first applies trajectory optimization with whole-body dynamics (Koenemann et al., 2015). The second approach optimizes for centroidal dynamics of a reduced-order model (Kajita et al., 2003; Apgar et al., 2018). Postural stability is crucial in motion planning; the trunk is underactuated, and its motions can be controlled only indirectly. With the control approaches above, the biped robots nowadays able to maintain an upright trunk and walk steadily on flat terrain (Kim et al., 2007; Sheng et al., 2013; Ding et al., 2018). However, sustaining trunk stability becomes difficult under external perturbations such as changes in ground level, since the control mechanism needs to regulate the additional change in the system's energy (Tokur, 2019). Perturbations can be either local, like a single step up/down, or global, as in up/downhill terrain.

Bipedal robots can counter terrain perturbations by modifying the ankle torque, hip torque, upper body rotation, and stepping patterns (Takenaka et al., 2009). The detailed strategy depends also on the capability of the robot to estimate its pose and the terrain. In the presence of an inclined terrain, many humanoid robots employ an ankle strategy, where the ankle pitch angle is adjusted based on the torso pitch angle feedback to prevent the robot from tilting [e.g., Nao (Ding et al., 2018), KHR-2 (Kim et al., 2007), SCUT-I (Sheng et al., 2013)]. Subsequent studies enhance the postural stability by increasing complexity in control: the biped robot SUBO-I adjusts its center of mass height by using a disturbance observer (Cho and Kim, 2018), Spring Flamingo adjusts the desired hip height of its virtual model controller (Chew et al., 1999), LOLA adapts its center of mass height based on contact force feedback (Sygulla and Rixen, 2020), and DRB-HUBO adapts its foot orientation (Joe and Oh, 2019) to traverse sloped surfaces and steps. On the other hand, some robots have vision-based perception, and therefore have extended capabilities to estimate the terrain and react to the changes (Fallon et al., 2015). These robots typically apply a task-level motion planning scheme that includes an optimizer for safe footholds, e.g., mixed-integer convex optimization for Atlas (Kuindersma et al., 2016), weighted *A*^⋆^ for Atlas and Valkyrie (Griffin et al., 2019). One common objective of these controllers is to consider trunk motion as undesired and try to maintain a fixed upright trunk throughout the motion. An exception is the SD-2 robot, which moves its trunk to offset the shift of its center of gravity due to the up/downhill slope (Zheng and Shen, 1990).

Bipedal running has an additional difficulty: large and rapidly changing ground reaction forces destabilize the underactuated trunk and the controller has less time to regulate the system during stance (Nilsson and Thorstensson, 1989; Gottschall and Kram, 2005). The essential properties of bipedal running are captured by the spring loaded-inverted pendulum model with a trunk (TSLIP). Within the TSLIP framework, virtual point (VP) control is proposed as a mechanism to achieve postural stability (Maus et al., 2008), which is implemented in the ATRIAS robot (Peekema, 2015) and the lower extremity exoskeleton LOPEZ II (Zhao et al., 2017) for walking gait. The VP approach forms a geometric coupling between the leg force and hip torque, based on the assumption that the ground reaction forces (GRF) intersect at a point above, at, or below the center of mass (CoM). The method is explored extensively for level walking (Sharbafi and Seyfarth, 2015; Lee et al., 2017) and level running (Andrada et al., 2014; Drama and Badri-Spröwitz, 2019, 2020; Sharbafi et al., 2012). However, there is no formalism to describe how VP control can be used to accommodate varying terrain conditions. So far, a single study conceptually suggests to offset the VP position horizontally and proportional to the change in step size to traverse stairs and slopes (Kenwright et al., 2011).

In this paper, we aim to explore model and controller parameterizations within the TSLIP-VP control framework to accommodate varying terrain conditions. In the first part of our work, we investigate whether the VP control mechanism can counteract external perturbations introduced by a single drop on the ground level. In the second part, we search for feasible ways to use the VP and achieve stable locomotion patterns for downhill running. The decrease in ground level adds energy to the system, equal to the change in potential energy. For the biped to maintain a constant speed, it is necessary to adjust the posture and leg parameters (i.e., leg length, leg stiffness, and damping, leg damping, leg angle at touch-down). We formalize which adjustments are sufficient for the TSLIP and the VP control scheme. Finally, we assess the VP as a method to constrain the GRF vectors and compare it to the friction cones that are commonly used in trajectory generation. The resulting insights can be used to efficiently parameterize control mechanisms that allow bipedal robots to compensate for ground level changes.

## 2. Materials and Methods

### 2.1. Related Work in Biomechanics

In order to extend the VP concept in a feasible and efficient manner, we take insights from human locomotion and analyze how humans cope with terrain changes. Humans adjust their leg properties and posture to respond to the changes in the ground level during running. In the presence of a visible single drop in ground level, humans adjust their leg parameters during the prior and at the perturbed step (Müller et al., 2012). They decrease their leg stiffness, increase their leg angle, and elongate their leg at touch-down (Müller and Blickhan, 2010; Müller et al., 2012). The peak GRF decreases at the preparation step, followed by an increased peak GRF at the perturbed step (Müller et al., 2012). The GRF vectors intersect at a virtual point below the center of mass (${\text{VP}}_{\text{B}}$), whose magnitude is reported as 30 cm for running over a ground level drop of 10 cm at 5 m s^−1^ (Drama et al., 2020). If the perturbation is visually hidden from the subjects (i.e., camouflaged), the adaptations in leg parameters are similar to those of the visible setting in principle, but display a larger behavioral variance between subjects. The vertical location of the estimated VP shows a larger variation for the camouflaged drop as well (Drama et al., 2020).

Terrain with a downhill slope can be modeled as a combination of subsequent ground level drops. The biomechanical literature for downhill running involves slopes up to −20 % and running speeds up to 5 m s^−1^ (Vernillo et al., 2017). In terms of temporal gait parameters, downhill running yields an increased aerial time, reduced step frequency, and decreased duty factor compared to level running (Vernillo et al., 2017). Human runners also adjust their postural orientation at heel strike to accommodate downhill terrain. The authors of Chu and Caldwell (2004) report two separate postural responses, where the first group of participants showed a more extended posture with low shock attenuation and the second participant group showed a more flexed posture with high shock attenuation.

Observation of the GRF patterns and the body's center of mass (CoM) energetics provides insights about the kinetic adaptations humans utilize for downhill running. The impact peak of the vertical ground reaction forces increases with the downhill slope, whereas the active peak either remains identical (Dick and Cavanagh, 1987; Gottschall and Kram, 2005; Telhan et al., 2010) or decreases (Wells et al., 2018). In addition, the maximum vertical GRF shifts from the active to the impact peak, as downhill slope increases (Wells et al., 2018). There are two different trends that are reported for the peak horizontal GRF during downhill running, which we summarize in Table 1. The authors of Dick and Cavanagh (1987), Gottschall and Kram (2005), and Wells et al. (2018) report an asymmetric gait behavior, where the peak propulsion forces become higher and peak braking forces become lower. Other studies (Yokozawa et al., 2005; Telhan et al., 2010) suggest that peak horizontal GRF remains the same. In downhill running, the external mechanical work (i.e., the work done to move the body's CoM with respect to the environment) is reported to be positive (i.e., energy generation) at shallow grades below −10.5 % and negative (i.e., energy dissipation) at steeper grades (Snyder and Farley, 2011).

**Table 1:** Downhill running experiments reported in the literature.

		**Peak vert. GRF**		**Peak horz. GRF**		
**Speed (ms^**−1**^)**	**Slope grade**	**Impact**	**Active**	**Braking**	**Propulsion**	**References**
4.5	−8.5 %	+14 %	No change	Double the	Half the	Dick and Cavanagh, 1987
			prop. force		braking force	
	3	+18 %		+27 %	-22 %	
3	−6°	+32 %	No change	+46 %	−40 %	Gottschall and Kram, 2005
	−9°	+54 %		+73 %	−61 %	
3,4,5	−3, −6, −9 %	Higher	–	No change		Yokozawa et al., 2005
3	−4°	Higher	No change	No change		Telhan et al., 2010
2.7	−2, −5, −8 %	Higher max vert. GRF		–		Lussiana et al., 2015
	−5 %	+14 %	−1 %	+2 %	+3 %	
4	−10 %	+32 %	−3 %	+2 %	No change	Wells et al., 2018
	−15 %	+47 %	−6 %	+10 %	−5 %	
	−20 %	+61 %	−8 %	+5 %	−13 %	

*Percentage values with a plus sign indicate an increase and a minus sign indicates a decrease in magnitude compared to the level running conditions. The slope percentage is calculated as the rise of the slope divided by its run times 100*.

### 2.2. Simulation Model

**Figure 1:**
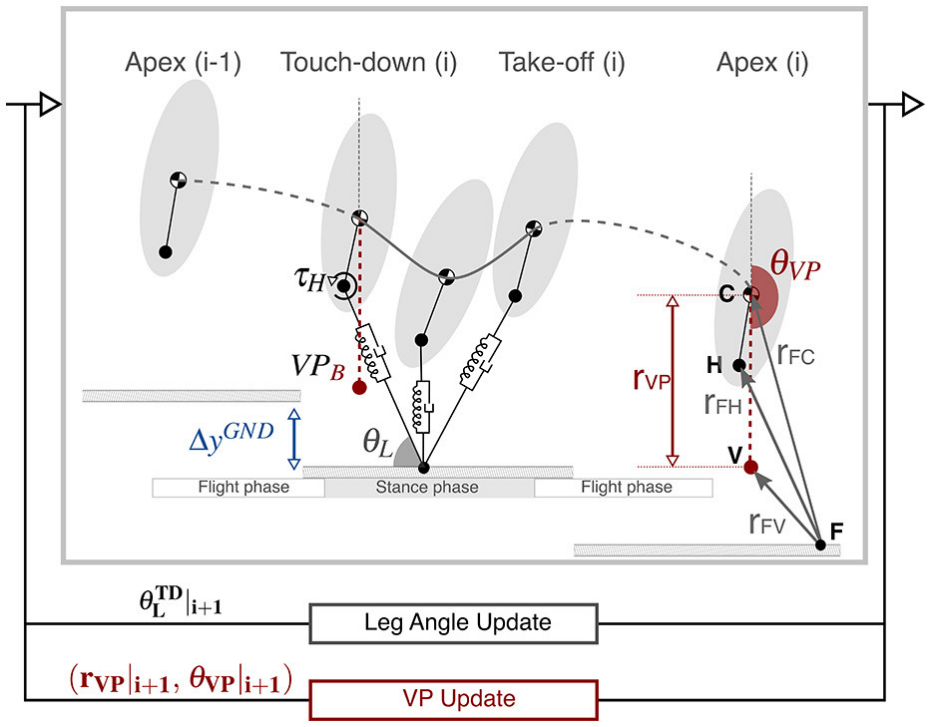
The TSLIP model has hybrid dynamics, which involve a flight phase followed by a stance phase. During the stance phase, the dynamics of the leg is passive, whereas the hip is actuated with a torque. The hip torque is defined in a way that the resultant ground reaction forces point to a virtual point. The virtual point is defined by a radius *r*_VP_ and angle θ_VP_. In our setup, we set the VP 30 cm below the center of mass (${\text{VP}}_{\text{B}}$), which corresponds to the value estimated for human running level terrain at a speed of 5 ms^−1^ (Drama et al., 2020). The VP angle is defined relative to a non-rotating frame, which has an origin at the center of mass and is aligned with the global vertical axis. In the Apex (i), the ${\text{VP}}_{\text{B}}$ is placed on the vertical axis passing through the center of mass, which corresponds to a VP angle of −180 °. The leg angle θ_L_ and VP angle θ_VP_ are updated at the end of each step. At each step, the ground level drops by Δ*y*^GND^ to simulate downhill running.

In this section, we describe the TSLIP model that we use to investigate the VP as a control scheme for accommodating ground level changes. The TSLIP model consists of a trunk with mass *m* and moment of inertia *J*, which is attached to a massless leg of length *l*, as shown in Figure 1. The morphological model parameters are selected from literature to match an 80 kg human with 1 m leg length, similar to Drama and Badri-Spröwitz (2019). The mean desired trunk angle ($\theta_{\text{C}}^{\text{DES}}$) is set to 10° forward, in accordance with Thorstensson et al. (1984) and Schache et al. (1999). The range of all model parameters and their selected values are provided in Table A1 of the supplementary file.

The leg consists of a parallel spring-bilinear damper mechanism, where the sum of spring (*F*_sp_) and damping (*F*_dp_) forces equal to the axial component of the GRF (_*F*_**F**_*a*_). The hip is actuated with a torque (τ_H_), which generates the tangential component of the GRF (_*F*_**F**_*t*_) expressed as,

(1) FFa=(k(l−l0)︷Fsp−cl(l−l0))︷Fdp×[−cosθL    sinθL]FFt=−l−1×FFa×[rFV×rFHrFV⋅rFH]×l︸τH×[    sinθL−cosθL].

The leg spring-damper jointly dissipates energy from the system, whereas the hip actuator supplies an equal amount of energy to preserve the energy balance. The hip torque is defined through a virtual point with radius (*r*_VP_) and angle (θ_VP_). Placing the VP above (${\text{VP}}_{\text{A}}$) or below (${\text{VP}}_{\text{B}}$) the center of mass affects the pattern of trunk angular motion^1^. The ${\text{VP}}_{\text{A}}$ is defined with respect to the *body frame*, which is centered at the CoM and is aligned with the trunk. On the other hand, ${\text{VP}}_{\text{B}}$ is defined with respect to the *world frame*, which is centered at the CoM and is aligned with the global vertical axis^2^ (Drama and Badri-Spröwitz, 2020).

The simulation starts at the apex state with zero vertical velocity, which is followed by a flight phase with ballistic dynamics. The initial state of the simulation is determined as the following: the apex height is set to ±10 % of the leg length depending on the speed (Geyer, 2005), the initial forward velocity to its desired value (2–5 ms^−1^), the initial trunk pitch angle to its desired mean value (10°), and the initial trunk pitch velocity is set to zero. The stance phase begins with the leg touch-down, during which the equations of motion for the CoM state (*x*_C_, *y*_C_, θ_C_) are expressed as,

(2) m[x¨Cy¨C]=FFa+FFt+g,   and Jθ¨C=−rFC×(FFa+FFt).

The stance phase ends when one of three conditions is met: the leg reaches its rest length *l*_0_, the vertical GRF becomes zero, or the vertical CoM velocity becomes zero after the mid-stance.

### 2.3. Proposed Control Method

**Figure 2:**
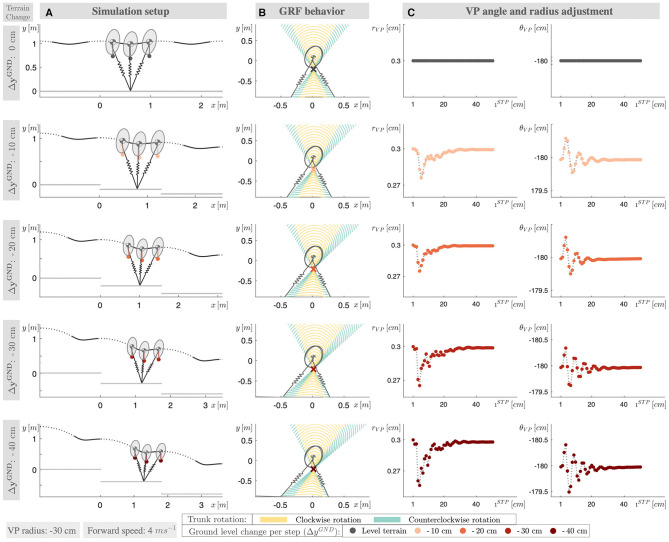
**(A)** The simulation setup for ground level change ranging from 0 to −40 cm ground level drop per step (Δ*y*^GND^). **(B)** The ground reaction force lines of the converged gaits (50th step) are shown with dotted lines, where the GRF lines corresponding a clockwise trunk rotation are shown in yellow color and, in teal color otherwise. The VP is marked with a cross. The distribution of the teal-yellow colored areas changes with the terrain grade, which corresponds the change in the trunk motion pattern. **(C)** The VP radius and angle modulation, as the gait reaches to its steady state condition.

The leg angle at touch-down $\theta_{\text{L}}^{\text{TD}}$, VP radius *r*_VP_, and angle θ_VP_ are linearly adjusted at the apex of each step, as in Figures 1, 2C. The purpose of the leg angle control is to achieve the desired forward speed and to assist in maintaining the desired trunk pitch angle. The control of VP angle modulates the system's energy indirectly by adjusting the coupling between the leg and hip. The control of the VP radius adjusts the rate of the change.

#### 2.3.1. Leg Angle Control

Conventional approaches regulate the leg angle based on the forward speed and its desired value (Raibert, 1986; Peuker et al., 2012; Sharbafi and Seyfarth, 2016). A recent study finds a correlation between the leg angle and the CoM height at apex based on human gait data (Seethapathi and Srinivasan, 2019). Drama and Badri-Spröwitz (2020) suggest to include the trunk pitch angle at the apex to leg angle controller function to bound the trunk oscillations in simulation. We combine all of these aspects in Equations (3b)–(3e) and adjust the desired leg angle at touch-down at each apex of step *i* as,

$$\begin{array}{ll} \theta_{\text{L}}^{\text{TD}}{|}_{\text{i}}=\theta_{\text{L}}^{\text{TD}}{|}_{\text{i}-1} & :\text{leg touch-down angle at previous step, (3a)}\\ \;+k_{y}\left(\Delta y_{\text{C}}^{\text{AP}}{|}_{\text{i}-1}^{\text{i}}+\Delta y^{\text{GND}}{|}_{\text{i}-1}^{\text{i}}\right) & :\text{difference in subsequent apex heights, (3b)}\\ \;+k_{{\dot{x}}_{0}}\left({\dot{x}}_{\text{C}}^{\text{DES}}-{\dot{x}}_{\text{C}}^{\text{AP}}{|}_{\text{i}}\right) & :\text{deviation of the apex forward speed from desired, (3c)}\\ \;+k_{\dot{x}}\left(\Delta {\dot{x}}_{\text{C}}^{\text{AP}}{|}_{\text{i}-1}^{\text{i}}\right) & :\text{difference in subsequent apex forward speeds, (3d)}\\ \;+k_{\theta }\|\theta_{\text{C}}^{\text{DES}}-\theta_{\text{C}}^{\text{AP}}{|}_{\text{i}}\| & :\text{deviation of the apex trunk pitch angle from desired, (3e)}\\ \;+k_{\bar{\theta }}\left(\theta_{\text{C}}^{\text{DES}}-{\bar{\theta }}_{\text{C}}^{\text{AP}}{|}_{\text{i}-1}^{\text{i}}\right) & :\text{deviation of the mean trunk angle from desired}.\text{ (3f)} \end{array}$$

where the controller gains (*k*_ẋ_, *k*_ẋ_0__) regulate the forward speed, the gains $\left(k_{\theta },k_{\bar{\theta }}\right)$ bound the oscillations of the trunk, and the gain *k*_*y*_ guides the stabilization in height. In our notation, Δ denotes the difference and superscript bar denotes the average value. If the terrain involves a downhill slope, we include the deviation of the mean trunk angle during stance from the desired trunk angle, which is expressed in Equation (3f). The leg angle controller gains are adjusted together with the leg damping coefficient, based on the criteria described in section 2.3.3. We provide the range for the controller gains in section 7.1. In the course of adjusting gains, we make sure to achieve the desired forward speed and mean trunk angle in a smooth fashion, while excluding non-periodic and period-n trajectories.

#### 2.3.2. Virtual Point Radius and Angle Control

We adjust the VP radius as a function of the angular velocity at leg take-off $\Delta {\dot{\theta }}_{\text{C}}{|}_{\text{t}=0}^{\text{TO}}$, and the VP angle based on the difference between the desired mean body angle $\theta_{\text{C}}^{\text{DES}}$ and mean body angle observed in the last step Δθ_C_ as,

(4a) $$\begin{array}{ll} r_{\text{VP}}{|}_{\text{i}} & =\{\begin{array}{ll} r_{\text{VP}}{|}_{\text{i}-1}+r_{\text{VP}}^{\prime } & \text{if }i=i_{\text{SD}}\\ \frac{r_{\text{VP}}{|}_{\text{i}-1}+\max\left(0,r_{\text{VP}}^{\text{DES}}-k_{r_{\text{VP}}}\|\Delta {\dot{\theta }}_{\text{C}}{|}_{\text{t}=0}^{\text{TO}}\|\right)}{2} & \text{otherwise} \end{array} \end{array}$$

(4b) $$\begin{array}{ll} \theta_{\text{VP}}{|}_{\text{i}} & =\{\begin{array}{ll} \theta_{\text{VP}}{|}_{\text{i}-1}+\theta_{\text{VP}}^{\prime } & \text{if }i=i_{\text{SD}}\\ \theta_{\text{VP}}^{\text{DES}}+k_{\theta_{\text{VP}}}\left(\theta_{\text{C}}^{\text{DES}}-\Delta \theta_{\text{C}}{|}_{\text{TD}}^{\text{TO}}\right) & \text{otherwise}. \end{array} \end{array}$$

The VP adjustment takes place at the end of the step, at apex. If there is a change in the ground level, the VP controller reacts to the changes with one step delay. This delayed response poses no problem for downhill running, since the model and control parameters are already tuned to compensate a step-wise continuous perturbation introduced by the global down-slope (see the example in Figure 2C). However, this is not the case for running over a terrain with a single step-down, where the control parameters are adjusted for flat terrain conditions. The sudden external perturbation might deviate the state excessively, if there is no appropriate response during stepping down. In particular at slow speeds, the trunk flexion/extension during the step-down (step *i*_SD_) might become too large with the increase in the stance time, and the controller might not recover the state in the following steps. To address this issue and reduce the angular rotation during step-down, we propose to offset the VP reference by $\left(r_{\text{VP}}^{\prime },\theta_{\text{VP}}^{\prime }\right)$ at the end of step *i* − 1 in Equation (4b).

#### 2.3.3. Gait Generation and Simulation Configuration

Our simulation study explores two different terrain conditions. In the first set of experiments, the terrain involves a single step-down perturbation. We conduct a parameter sweep spanning step-down heights of Δ*y*^STP^ = [−10, −20, −30, −40] cm and speeds of ẋ_C_ = [2, 3, 4, 5] m s^−1^. We perform the sweep for both VP above (${\text{VP}}_{\text{A}}$) and below (${\text{VP}}_{\text{B}}$) the CoM, where we set the VP radius to 30 cm based on Drama et al. (2020).

In the second set of experiments, we simulate a downhill slope by deceasing the ground level by a constant amount of (Δ*y*^GND^) at the apex of each step. The slope of the terrain depends on the running speed, which is provided in Table 2. For downhill running, we focus on using ${\text{VP}}_{\text{B}}$ as the control target, as it is the behavior that is observed in human running. While it may be possible to adjust additional model parameters and the control strategy to use ${\text{VP}}_{\text{A}}$, we found the ${\text{VP}}_{\text{A}}$ to be unstable and difficult to parameterize. The ${\text{VP}}_{\text{A}}$ tends to work against the trunk flexion when stepping down, while ${\text{VP}}_{\text{B}}$ assists to the natural response.

**Table 2:** Terrain slope corresponding to the ground level change per step Δ*y*^GND^ and running speed for ${\text{VP}}_{\text{B}}$ gaits.

**Running**	**Ground level change per step (Δy^GND^)**			
**Speed (ms**^**−1**^**)**	−10 cm	−20 cm	−30 cm	−40 cm
2	7.2°	12.1°	16.8°	21.5°
3	5.3°	9.0°	12.4°	15.4°
4	4.4°	7.3°	9.7°	12.4°
5	4.0°	6.6°	8.6°	10.5°

**Figure 3:**
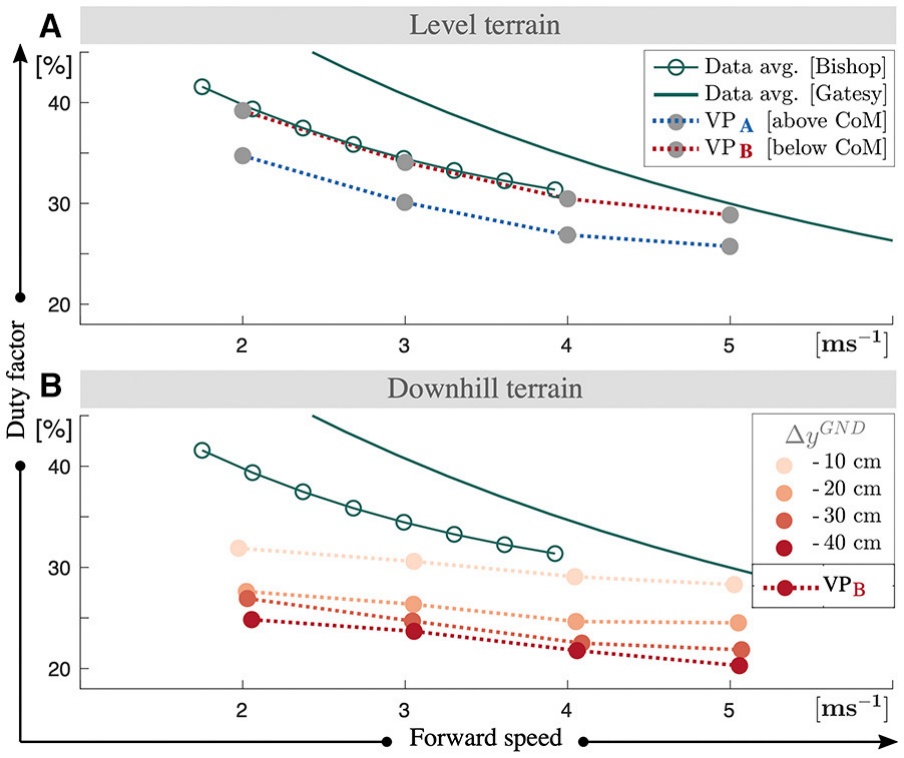
Duty factor values of the gaits for the level **(A)** and downhill **(B)** terrain conditions. If the terrain involves a single step-down, the gaits yield duty factor values equal to the duty factor values at level terrain. We tune the controller parameters and the damping coefficient, in a way that the resultant gaits yield duty factors similar to ones observed in human running. We also preserve the functional relation that the duty factor decreases with increasing running speed. For downhill running the duty factor values get lower and the decrease is proportional to the downhill grade.

To adjust the damping coefficient *c*, we use the duty factor as the primary criteria. Duty factor equals to the stance time over the stride and it decreases with the running speed in human running, as shown with green lines in Figure 3 (Gatesy and Biewener, 1991; Bishop et al., 2017). We impose the same relation when tuning our gaits for level running, where the duty factor of our level running gaits range between 40–28% for ${\text{VP}}_{\text{A}}$ and 35–25% for ${\text{VP}}_{\text{B}}$ (see Figure 3A). When the terrain has a single step-down, the control scheme attenuates the perturbation and brings the system back to its initial equilibrium state to the same duty factor level. In case of a downhill terrain, the duty factor decreases proportional to the terrain grade and ranges between 32–20% for ${\text{VP}}_{\text{B}}$, which is shown in Figure 3B. A lower duty factor indicates an increase in peak vertical GRF, which can be prevented with additional parameter adjustments such as decreasing the leg stiffness.

**Figure 4:**
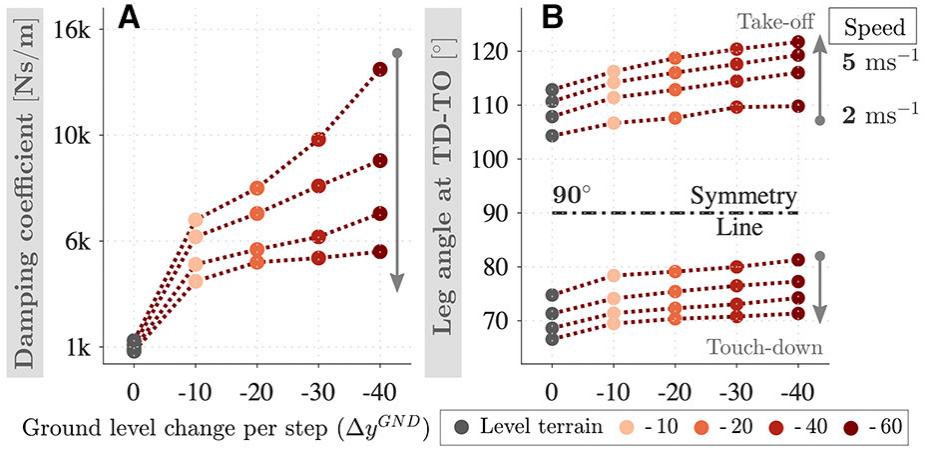
Leg damping coefficient **(A)** and the leg angle at touch-down/take-off events **(B)** corresponding to downhill running of speeds 2–5 ms^−1^ and gradients 0–40 cm per step. Damping coefficient and leg angle at touch-down decrease with the speed and increase with the terrain grade.

The second criteria we consider is related to the take-off conditions. If the damping coefficient is too large, the vertical CoM acceleration becomes zero before the leg reaches to its rest length or the vertical GRF reaches to zero. The stance phase is terminated early with GRF suddenly cut-off, and take-off to apex phase of the respective step does not happen. To avoid this unrealistic scenario, we limit the maximum value of the damping coefficient. Given these considerations, we obtain the damping coefficients in Figure 4A, which decrease with speed and increase by a factor of 5–8 with the terrain grade. The leg angle touch-down exhibits a similar relation to leg damping coefficient, where it decreases with the speed and increases 4–7% with the terrain grade, as shown in Figure 4B.

In case of the single step-down, the control approach rejects the perturbation in the following level-terrain steps, and returns the system to its initial equilibrium. At the downhill terrain, the controller finds a new equilibrium with step-wise disturbance rejection. At increasing terrain slope, we observe an increase in the asymmetry of the gait patterns (see Figures 2A,B, an asymmetry in the CoM trajectory and GRF).

## 3. Simulation Results

In this section, we show how our VP controller responds to the changes in the ground level. We describe the kinetic properties of the gaits and the work distribution between the leg and hip.

### 3.1. Terrain With a Single Step-Down

**Figure 5:**
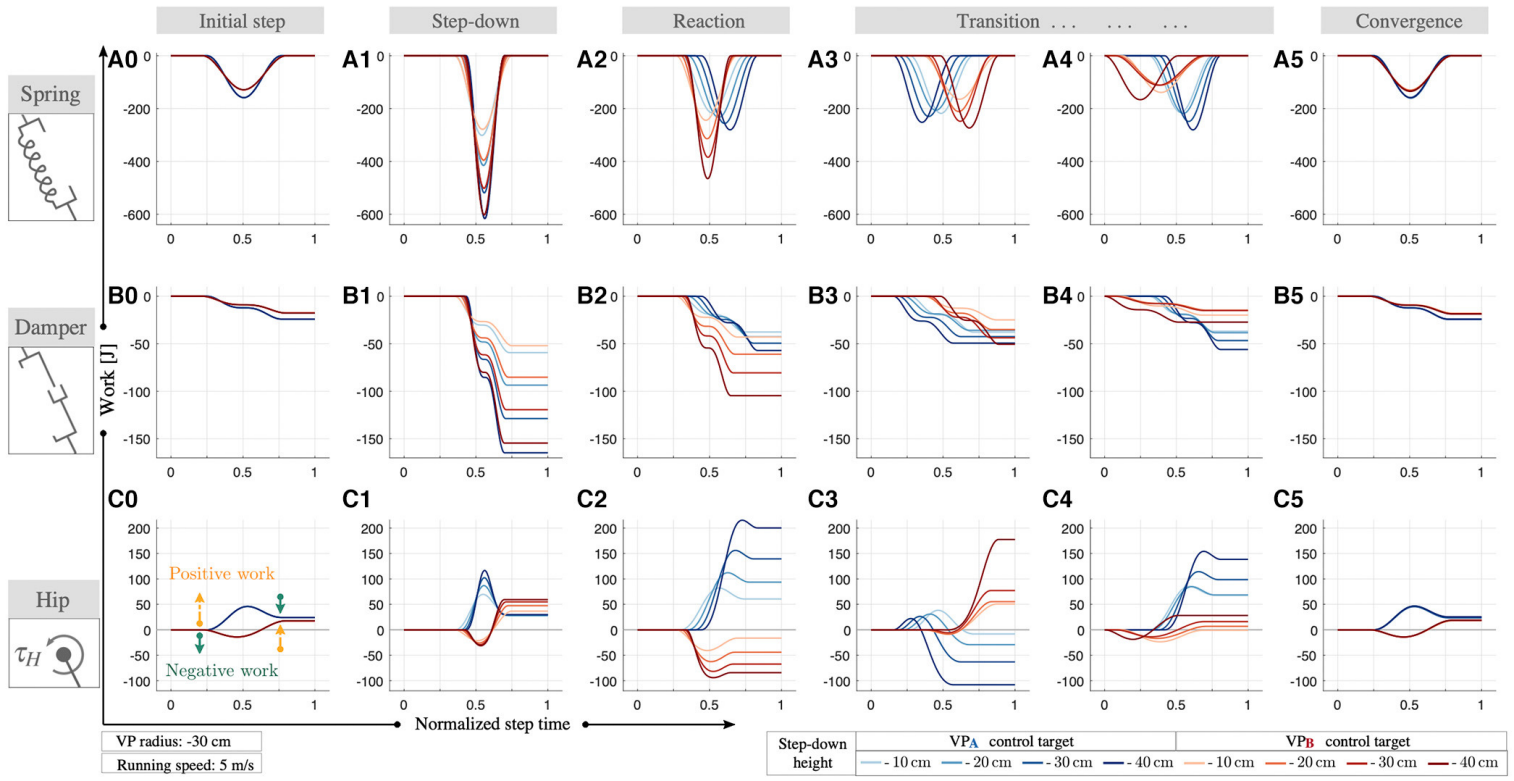
The work performed by the leg spring **(A)**, leg damper **(B)**, and the hip actuator **(C)** during running at 5 ms^−1^ for both ${\text{VP}}_{\text{A}}$ and ${\text{VP}}_{\text{B}}$ control targets with 30 cm radius. The energy of the system increases with the step-down. The maximum leg compression increases, which leads to an increase in the energy stored/recoiled by the leg spring **(A1)** and the energy dissipated by the leg damper **(B1)**. The net energy generated by the hip increases as well **(C1)**. In the next step, the VP control starts reacting to the changes in state **(A2–C2)**. In the subsequent steps, the net leg work gradually decreases **(B2–B4)**, whereas the net hip work alternates its sign to regulate the excess energy **(C2–C4)**. Both ${\text{VP}}_{\text{A}}$ and ${\text{VP}}_{\text{B}}$ control targets are able to attenuate the excess energy introduced by the step-down and bring the system back to its initial equilibrium conditions within 50 steps.

The initial step before the step-down is in equilibrium state for level-terrain, where the leg removes energy from the system and the hip supplies an equal amount of energy, as shown in Figures 5A0–C0 for 5 m s^−1^ speed. The energy provided by the hip actuator depends on the position of the VP. When the control target is ${\text{VP}}_{\text{A}}$, the hip produces energy at early stance and dissipates energy partially after mid-stance, which results in a net positive work that is required to counterbalance the leg damper (blue lines in Figure 5C). Conversely, the hip actuator with ${\text{VP}}_{\text{B}}$ control target dissipates energy first and generates a large amount of energy afterwards to compensate for both the prior loss and leg damping (red lines in Figure 5C).

At step-down perturbation, the total energy of the system increases proportional to the step height, which disrupts the energy balance of the system. Since the perturbation is one-time-only, the controller has the opportunity to dissipate the perturbation in the following multiple steps, unlike downhill running where the perturbation is continuous and needs to be dissipated within a single step. In both cases, the additional energy can not be converted to kinetic energy, since the leg angle controller attempts to maintain a constant running speed and constant trunk angular excursion. As a consequence, the excess energy needs to be dissipated through the interplay between in the leg and the hip.

During the step-down, the maximum leg compression increases by a factor of 1.3–2 for ${\text{VP}}_{\text{A}}$ and 1.5–2.2 for ${\text{VP}}_{\text{B}}$, which leads to an increase in the energy stored/recoiled by the spring by a factor of 1.9–3.8 for ${\text{VP}}_{\text{A}}$ and ${\text{VP}}_{\text{B}}$ (see Figure 5A1). Alongside the spring, the energy dissipated by the leg damper increases by 6–11 times for ${\text{VP}}_{\text{A}}$ and 7–13 times for ${\text{VP}}_{\text{B}}$ (see Figure 5B1). Both the energy stored/recoiled by spring and the energy dissipated by damper increase with step-down height and running speed. The net hip work increases by a factor of 5.1–5.2 for ${\text{VP}}_{\text{A}}$ and 6–7.3 for ${\text{VP}}_{\text{B}}$. In addition, the peak positive hip work gets 3.6–4.6 times higher for ${\text{VP}}_{\text{A}}$, whereas the peak negative hip work is 4.6 times larger for ${\text{VP}}_{\text{B}}$ (see Figure 5C1). The VP position update takes place at the end of the step-down, and the controller reacts to the changes in the state in the next steps. In the following steps, we observe leg and hip energy fluctuations, where the net damping energy decreases (see Figures 5B2–B4) and net hip energy alternates its sign over the subsequent steps (see Figures 5C2–C4). The energy stored/recoiled by the leg spring decreases at each following step towards its equilibrium value (see Figures 5A2–A4). In addition, we observe a temporal shift in the stance phase, which alternates over the course of the transition period.

We report gait parameter combinations for both ${\text{VP}}_{\text{A}}$ and ${\text{VP}}_{\text{B}}$ approaches, where the controllers are able to bring the system back to its initial equilibrium conditions within 50 steps (see Figures 5A5–C5). The extended results for speeds 2–4 ms^−1^ can be found in Figure A1 of the supplementary file.

### 3.2. Downhill Terrain

In downhill running the biggest challenge is to reject the energy introduced by the ground level change within a single step. The controller needs to bring the system to a new equilibrium, where the energy increase due to step-down is dissipated within a single stance phase.

**Figure 6:**
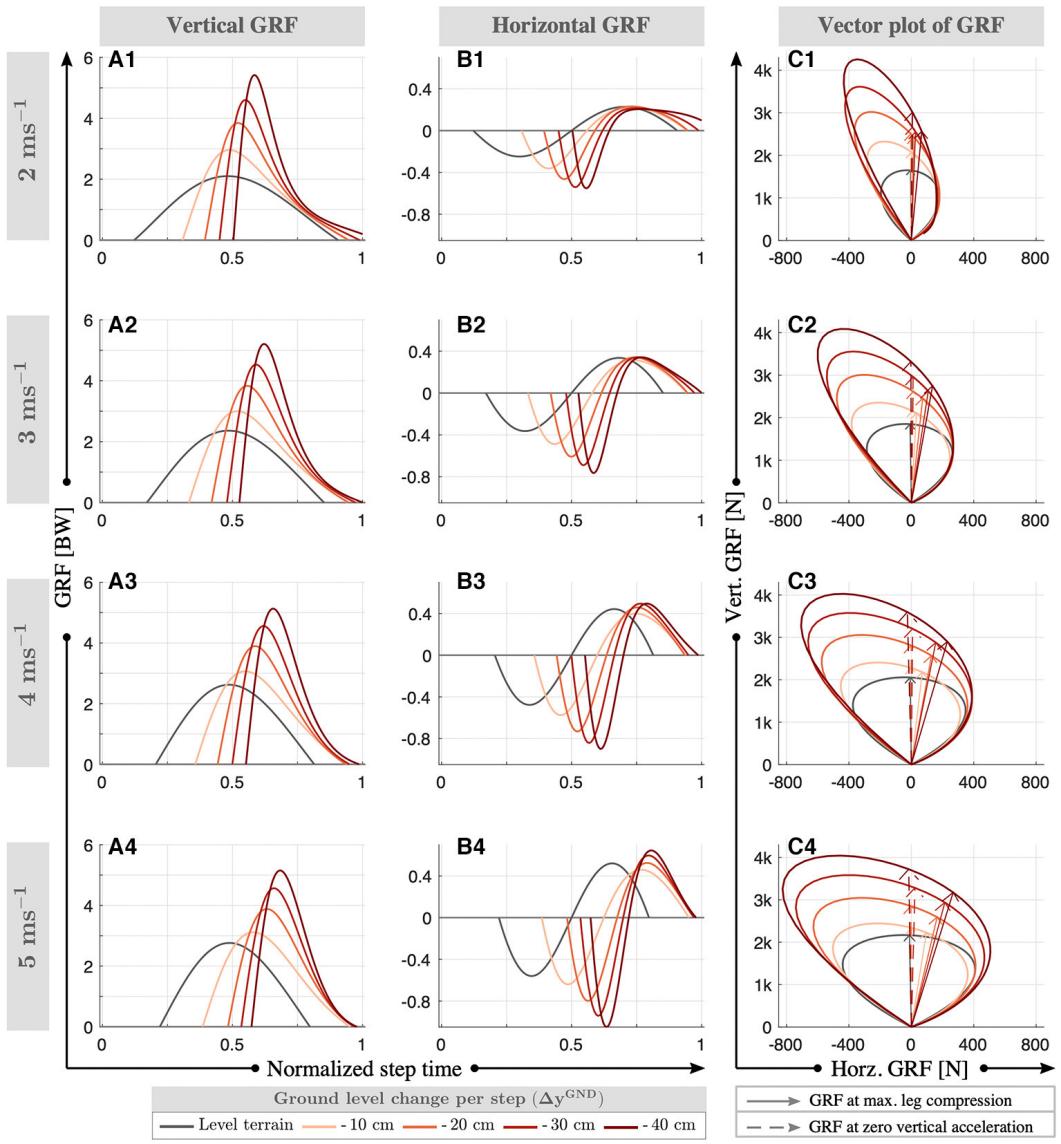
Normalized vertical **(A)** and horizontal **(B)** ground reaction forces for downhill running with ${\text{VP}}_{\text{B}}$ control target at speeds of 2–5 ms^−1^. The vector plot is provided in **(C)**, where the GRF vector at maximum leg compression and zero vertical acceleration (min. vertical velocity of the CoM) are shown with solid and dashed arrows, respectively. The peak vertical GRF increase with speed and increasing terrain grade. The peak braking forces (min. horizontal GRF) and peak propulsion forces (max. horizontal GRF) show a similar behavior. An exception is the peak propulsion forces at Δ*y*^GND^ = - 10 cm **(B1)**, which decrease with respect to the level terrain conditions. In addition, the stance phases shift toward the end of the step and the GRF profiles become more left-skewed with higher terrain grade.

**Figure 7:**
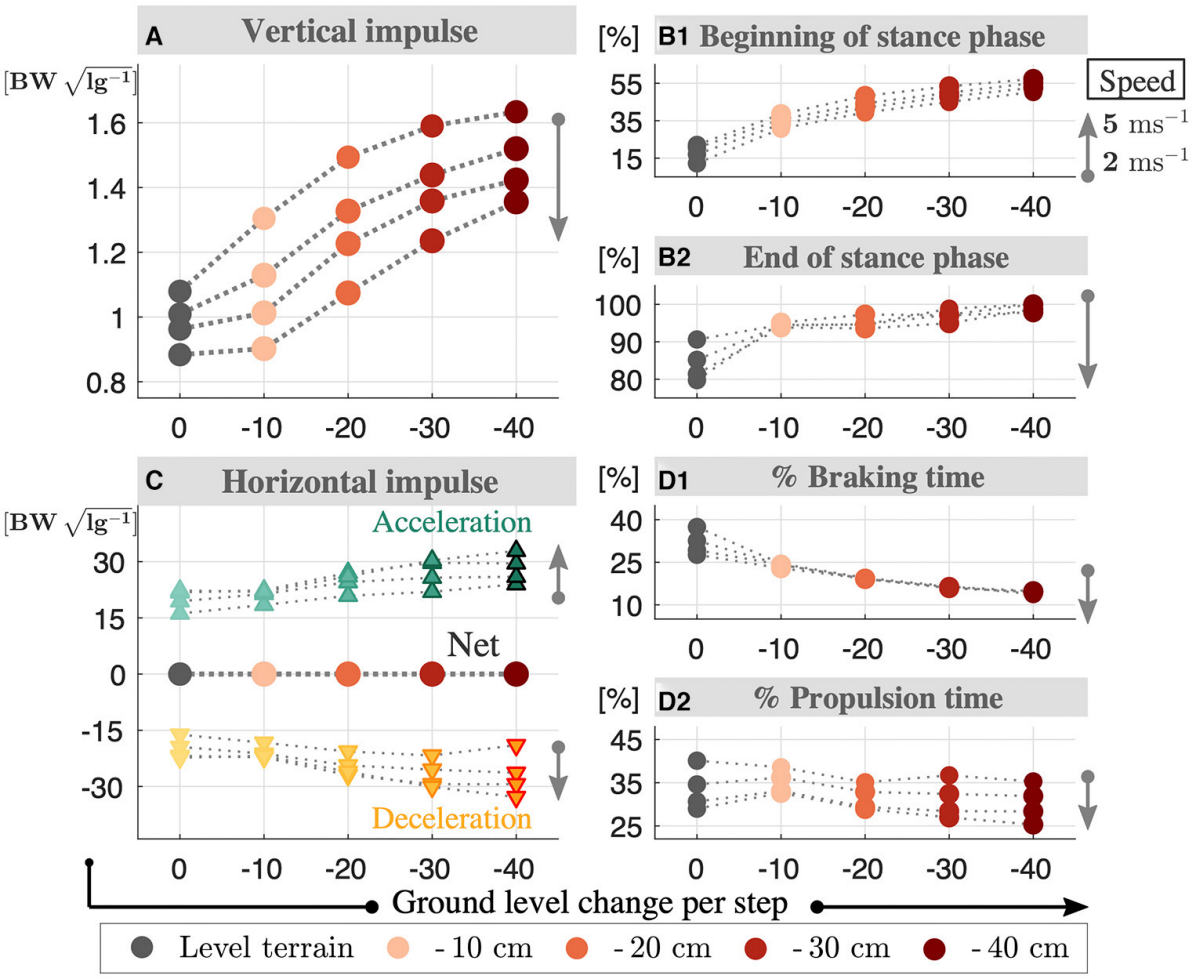
Normalized vertical **(A)** and horizontal **(C)** impulses for downhill running with ${\text{VP}}_{\text{B}}$ control target at speeds of 2–5 ms^−1^. The vertical impulse increases with terrain grade and decreases with speed. Both braking and propulsion impulses increase with the terrain grade and speed, where the sum is equivalent to zero. The temporal analysis is provided in the right column, where the beginning **(B1)** and end **(B2)** of stance phase over step time is presented in %, as well as the times spent on braking **(D1)** and propulsion **(D2)** intervals. The stance time is shifted toward the end of step and braking/propulsion intervals get shorter, as the terrain grade increases.

To characterize the new equilibrium conditions corresponding to different downhill grades, we evaluate the GRF profiles and impulses. The peak vertical GRF depends on the running speed and increases from 2 to 2.7 body weights as the speed rises from 2 to 5 ms^−1^ in level running (gray lines in Figure 6A). At downhill terrain, the peak vertical GRF increases by a factor of 1.2–2.5 proportional to terrain grade, which reaches up to 5.4 body weights (see Figures 6A1–A4). The impulses corresponding to the vertical GRF are quantified in Figure 7A, which range between 0.9 and 1.1 for level running and increase from 0.9–1.3 to 1.3–1.6 with the terrain grade. In addition, we observe left-skewed vertical GRF profiles, where the asymmetry becomes more pronounced as the terrain grade increases.

The literature in human running provides different answers on how the horizontal GRF responds to downhill terrain conditions. Studies by Dick and Cavanagh (1987), Gottschall and Kram (2005), and Wells et al. (2018) observe an increase in peak braking forces (min. horizontal GRF) and a decrease in peak propulsion forces (max. horizontal GRF), whereas Telhan et al. (2010) and Yokozawa et al. (2005) report no changes. In our level gaits, the peak horizontal GRF magnitude increases with speed and range between 0.22 and 0.56 body-weights. At downhill terrain, the peak braking force increases by a factor of 1.1–2.2 with the terrain grade (see Figures 6B1–B4). The peak propulsion forces decrease by a factor of 0.96–0.88 when the terrain grad is Δ*y*^GND^ = - 10 cm, where they increase by a factor of 1.0–1.3 for higher grades. This dependence on terrain grade could possibly be related to the metabolic minimum observed in 20 % downhill grade in human running (Minetti et al., 2002; Vernillo et al., 2017), but no relevant experimental data exists yet. The peak braking forces being larger than the peak propulsion forces raises the question whether the net horizontal GRF impulse is negative valued to compensate the downhill conditions. We see in Figure 7C that this is not the case. Both the braking and propulsion impulse becomes 1.0–1.5 higher with terrain grade, while the sum remains zero. In other words, there is no net horizontal acceleration in our downhill running gaits. In addition, we observe a left-skew in the braking and propulsion force patterns, similar to the vertical GRF profiles.

To analyze the asymmetric behavior that we observe in the GRF patterns, we analyze the gait's horizontal and vertical impulses in Figures 7B–D. In level running, the stance phase begins at 12–11% of the step and ends at 90–79%, where the braking/propulsion intervals comprise 37–27 and 40–29% of the step time, respectively. As the downhill terrain grade increases, the stance phase shifts toward the end of step, while the braking/propulsion intervals decrease. For the grade Δ*y*^GND^ = −40 cm, the stance phase starts at 55–57% and continues until the end of the step time. In this case, the phase between leg take-off and the apex diminishes.

**Figure 8:**
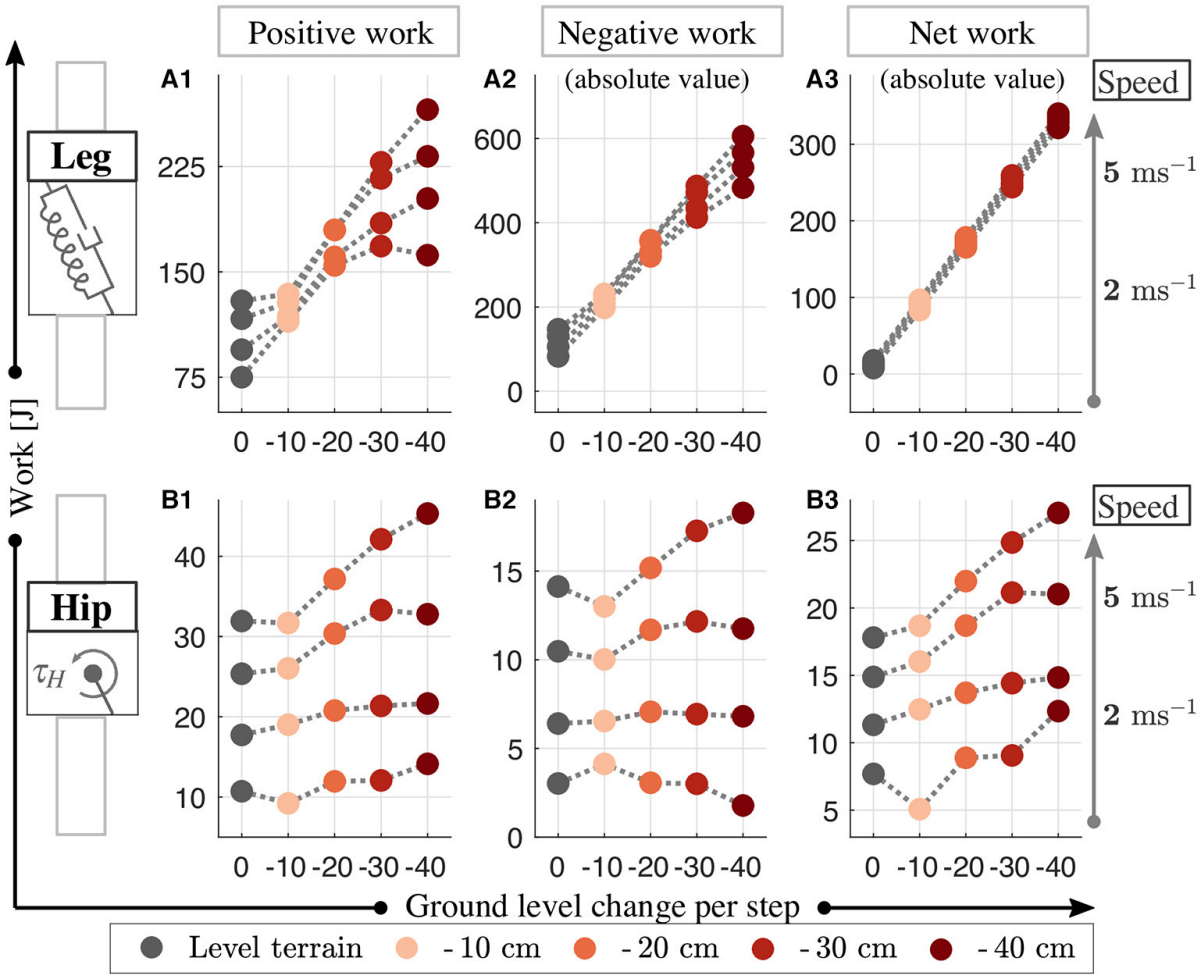
The positive **(A1,B1)**, negative **(A2,B2)**, and net **(A3,B3)** work performed by the leg and hip for downhill running with a ${\text{VP}}_{\text{B}}$ control target at speeds of 2–5 ms^−1^. The increase of energy caused the downhill terrain is compensated by the increase in the energy dissipated in leg and the energy generated by hip increases.

Next, we investigate how the VP control compensates for the additional energy caused by the ground level changes. Unlike Kenwright et al. (2011) suggests offsetting the VP position horizontally, we found it sufficient to increase the damping coefficient to accommodate downhill grades. The time progression of the work performed by the leg and hip is provided in Figure A2 of the supplementary file and corresponding numerical values for the positive/negative/net works are shown in Figure 8. As the terrain grade increases, the positive leg work increases by a factor of 1.5–2.2 (see Figure 8A1), negative leg work by a factor of 1.6–6 (see Figure 8A2), and the net leg work by a factor of 5.5–37 (see Figure 8A3). On the other hand, the positive hip work gets 1.02–2.4 times higher (see Figure 8B1), negative hip work gets 1.01–1.3 times higher with the exception of Δ*y*^GND^ = - 10 cm (see Figure 8B2), and net hip work gets 1.1–1.5 times higher with the terrain grade (see Figure 8B3).

**Figure 9:**
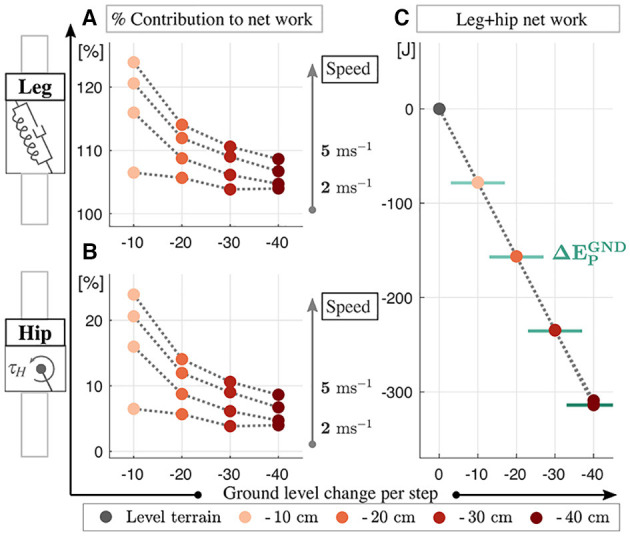
Work contribution of the leg **(A)** and hip **(B)** to the net work (in percent) and the numerical values of the net work corresponding to varying ground level changes **(C)**. The leg removes energy from the system, whereas the hip injects energy. The amount of potential energy added to the system at the downhill conditions ($\Delta E_{\text{P}}^{\text{GND}}$) is marked with solid green lines in **(C)**. We see that the increase from additional potential energy is fully compensated by the leg-hip actuators.

In Figure 9, we look at the distribution of the net work provided by the leg and hip actuators. The sum of the energy dissipated by the leg (see Figure 9A) and produced by the hip (see Figure 9B) amounts to 100 %. When the terrain grade increases, the percentage contributions of the leg and hip decrease. The combined work of the leg and hip dissipates the energy introduced by the terrain's potential energy difference completely (see $\Delta E_{\text{P}}^{\text{GND}}$ in Figure 9C). The relation holds for all terrains tested, and for all running speeds with the exception of the 2 m s^−1^ gait at terrain grade of Δ*y*^GND^ = −40 cm (see dark red marker in Figure 9C). In this case, the energy discrepancy is due to the early leg take-off, where the stored spring energy recoils completely.

## 4. Discussion

### 4.1. The Virtual Point in Relation to the Friction Cone

We can think of the VP concept as a method to bound the GRF vectors, which is analogous to the concept of friction cones in robotics. In this context, the vertical position of the VP determines the bounds of the cone, where the cone becomes wider as the VP is placed closer to the ground level. Consequently, placing the VP above/below the CoM can be associated with a narrow/wide cone, respectively.

For level running, the VP method generates GRF vectors that sweep a symmetric cone in counter-clockwise direction throughout the stance phase, as shown with gray lines in Figure 6C. When the GRF vector becomes vertical, the leg reaches its minimum length (solid arrow) and the vertical acceleration of the CoM reaches to zero (dashed arrow). The GRF vectors span a wider cone as the running speed increases, which implies that a larger friction coefficient (μ) is necessary to run at high speeds. The half-angle of the cone (β = arctan μ) increases from 18 to 28° as the running speed increases from 2 to 5 m s^−1^.

When running over a single step-down, the perturbed step displays sine-shaped GRF pattern without any skew (i.e., no phase shift). However, braking and propulsion parts of the horizontal GRF display asymmetric peaks and have different time span, since the perturbed step is not in equilibrium. The behavior of the GRF vectors in the subsequent transition steps depends on the VP and leg angle adjustment and alternates at each step. Therefore, we are not able to draw any further insights.

For downhill running, the VP method generates GRF vectors that sweep an asymmetric cone throughout the stance phase, as shown with red lines in Figure 6C. We observe that the left half of the cone is narrower compared to the right half, and contains the majority of the high magnitude GRF vectors.

As the terrain grade increases, the left half of the cone becomes narrower and the right half wider, which is quantified in Table 3 for min./max. terrain grades. In other words, the asymmetry increases with the terrain grade. As the running speed increases, the GRF vectors span a wider cone, similar to level running. When the GRF vector becomes vertical, the vertical acceleration of the CoM reaches to zero (dashed arrow). Opposed to level running, the maximum leg compression occurs later in stance phase, which has implications when defining the mid-stance of the gait. Typically, the mid-stance is defined as the point where vertical CoM acceleration becomes zero or where the leg compression is maximum. These two events coincide for level running, and therefore the mid-stance can be defined either way. However, these events occur at different times for downhill running, and therefore a distinction becomes necessary when defining the mid-stance.

**Table 3:** Boundary angles corresponding to the left/right half of cones generated by the GRF vectors.

	**Ground level change per step (Δy^GND^)**			
**Running**	**Δ*y*^GND^ = - 10 cm**		**Δ*y*^GND^ = - 40 cm**	
**speed (ms**^**−1**^**)**	**Left half cone (°)**	**Right half cone (°)**	**Left half cone (°)**	**Right half cone (°)**
2	14	21	10	26
3	19	28	15	33
4	22	31	19	37
5	24	34	22	40

*The angles are quantified for downhill terrain with ground level change per step Δy^GND^[−10, −40]cm*.

The gaits we generated span a friction cone with coefficients μ ∈ [0.32 0.53] for level and μ ∈ [0.17 0.83] for downhill terrain, which is in agreement with the values used in legged robots [μ ∈ [0.14 0.9] (Kajita et al., 2004; Caron et al., 2015; Brandäo et al., 2016; Fahmi et al., 2019)]. In legged robotics, the friction cone is typically included in the optimization constraints, which does not guarantee the smoothness and continuity of the resultant GRF vector sequence. On the other hand, the GRF vectors of the VP approach sweep a cone with a smooth change in magnitude. Moreover, in case of downhill running, the VP method provides insights into how the GRF vectors can be asymmetrically constrained. Therefore, employing different VP targets has a potential to adapt terrain with different friction in the field of humanoid robots. For instance, if the surface has a low friction (e.g., icy terrain), we can raise the VP position up to impose a narrower bound for the GRF and avoid feet slippage.

### 4.2. Challenges of Implementing a Virtual Point Control

Previous work suggested that the VP control could be beneficial in robotic applications, where the VP location can be modified to trade-off between the energy required for the leg and hip joints (Drama and Badri-Spröwitz, 2019). Our current work corroborates this by showing that the VP control can stabilize the posture and gait for single step-down perturbations and downhill terrain.

The challenge of implementing a VP based controller to a real robot will likely arise from the inaccuracies in the model and state estimation. VP controller requires estimates of the trunk state and measurements of the effective leg^3^ force. State estimation for legged robots is difficult, as its rapidly changing dynamics require robust, low-latency, and high-frequency state estimates (Camurri et al., 2020). Proprioceptive sensors such as IMUs, force/torque sensors and joint encoders are able to meet these requirements, but suffer heavily from sensor drift. The majority of bipedal robots use IMUs together with filter-based state estimation techniques to estimate its own trunk state (Hubicki et al., 2016). Paiman et al. (2016) proposes a VP based observer for a wearable robotic device, which uses an IMU with an unscented Kalman filter (UKF) to estimate trunk orientation, gyros to estimate trunk angular velocity, and an accelerometer to estimate linear CoM acceleration. Recent studies improve the state estimation accuracy by sensor fusion, and include exteroceptive sensors such as cameras and LIDAR (Wisth et al., 2019; Camurri et al., 2020).

Legged locomotion involves collision and rapidly changing dynamic forces, which makes force sensing challenging. The force/torque measurement capabilities depend on the robot's leg and actuator design. Conventionally, forces and torques are measured through dedicated force/torque sensors that are mounted proximally or distally (Semini et al., 2011), but stiff and heavy sensors manage rapid and harsh impacts not well. Robots with a series elastic actuators avoid direct force sensing and calculate the force indirectly by measuring the deflection of the compliant element (Pratt and Krupp, 2004; Renjewski et al., 2015). However, the presence of physical compliance places limitations on the bandwidth of the system. Recently, proprioceptive sensing estimates the force through the motor's current, which works well in combination with low gear ratios and a light-weight leg design (Seok et al., 2012; Wensing et al., 2017), and enables accurate force estimation even in the presence of high impacts (Grimminger et al., 2020).

ATRIAS robot walks and maintains postural stability applying a VP controller. The robot estimates its trunk's state with a high-precision IMU, and estimate the force with its high-resolution encoders on motors and compliant joints (Peekema, 2015). To implement our proposed VP controller for running with robot hardware, we propose IMUs, and filtering for estimating the trunk state, and proprioceptive actuation for leg force sensing and control.

## 5. Conclusion

In this work, we investigated the virtual point control mechanism in its ability to cope with single step-down perturbations and at downhill terrains, using a spring inverted pendulum model with trunk. We showed that placing the virtual point either above or below the center of mass allows rejecting the perturbation caused by a single step-down in terrain up to a step height of 40 cm at speeds of 2–5 ms^−1^. In addition, we found that increasing the leg damping and placing the virtual point below the center of mass is sufficient to compensate for the energetic and dynamic changes introduced by downhill running. No further virtual point manipulation is necessary. Our results provide an easy recipe to parameterize humanoid robot controllers, to adjust for varying terrain conditions.

## Data Availability Statement

The kinetic and kinematic data corresponding to the simulation gaits is provided as a Matlab structure in “data.mat” file, which can be found in Supplementary Materials. For further details regarding the data structure, please refer to the “data_info.md” document.

## Author Contributions

ÖD devised the project, developed the simulation framework, and analyzed the simulated gaits. AB-S supervised the project and provided critical feedback. ÖD and AB-S discussed the results and contributed to writing the manuscript. All authors contributed to the article and approved the submitted version.

## Conflict of Interest

The authors declare that the research was conducted in the absence of any commercial or financial relationships that could be construed as a potential conflict of interest.

## Footnotes

^1^The virtual points that are below the CoM and below the leg axis are referred to as VP_BL_ in Drama and Badri-Spröwitz (2019); Drama and Badri-Spröwitz (2020). In this paper, the ${\text{VP}}_{\text{B}}$ radius is −30 cm and is always below the leg axis.

^2^The body frame translates with the CoM and rotates with the trunk. The world frame translates with the CoM and does not rotate.

^3^The effective leg is the simplified leg defined between the hip joint and center of pressure.
