# Supplementary material for: Virtual Point Control for Step-Down Perturbations and Downhill Slopes in Bipedal Running
Source: Front Bioeng Biotechnol. 2020 Dec 18;8:586534. doi: 10.3389/fbioe.2020.586534 (PMC7775500; doi:10.3389/fbioe.2020.586534)
Supplement: Supplementary file 1 [file Data_Sheet_1.pdf]

Virtual Point Control for Step-down Perturbations and Downhill Slopes in Bipedal Running

Appendix

A1. TSLIP Model and Control Parameters

The model parameters of the TSLIP model are chosen from literature to match a human with $80\,\mathrm{kg}$ body mass and $1\,\mathrm{m}$ leg length, similar to Drama and Badri-Spröwitz (2019). We present parameter ranges from literature and our values selected here Table A1, which is recreated from Drama and Badri-Spröwitz (2019). We also provide the range for the leg angle controller gains.

Table A1: Model parameters of the TSLIP model corresponding to a human, and the range of the leg angle controller gains.

**Model Parameters**					
Name	Symbol	Units	Literature	Chosen	Reference
mass	m	kg	60-80	80	Sharbafi et al. (2013); Sharbafi (2017)
moment of inertia	J	$\mathrm{kg\,m^2}$	5	5	Sharbafi et al. (2013); de Leva (1996)
leg stiffness	k	$\mathrm{kN\,m^{-1}}$	16-26	18	Sharbafi et al. (2013); McMahon and Cheng (1990)
leg length	l	m	1	1	Sharbafi et al. (2013); Sharbafi (2017)
leg angle at touch-down	θ_L^{TD}	°	78-71	$f_H(\dot{x})$	Sharbafi et al. (2013); McMahon and Cheng (1990)
distance Hip-CoM	r_{HC}	m	0.1	0.1	Sharbafi et al. (2013); Wojtusch and von Stryk (2015)

**Leg Angle Controller Gain Range**					
Terrain type and VP control target	$k_{\dot{x}}$	$k_{\dot{x}_0}$	k_y	k_θ	$k_{\dot{\theta}}$
step-down with $\mathrm{VP_A}$	[0.08 0.17]	[0 0.15]	[-0.05 0]	[0 0.46]	[0 0.15]
step-down with $\mathrm{VP_B}$	[0.1 0.3]	[0.03 0.18]	[-0.05 0]	[0 0.02]	[-0.01 0.15]
downhill with $\mathrm{VP_B}$	[0.1 0.3]	[0.05 0.2]	[-0.05 0]	[0 0.07]	[0.025 0.15]

A2. Terrain with a Single Step-down

Here we present our results for single step-down perturbations, extended to running speeds of 2-5 m s^{-1}. The work performed by the leg spring, damper and the hip are given in Figure A1 for $\mathrm{VP_A}$ and $\mathrm{VP_B}$.

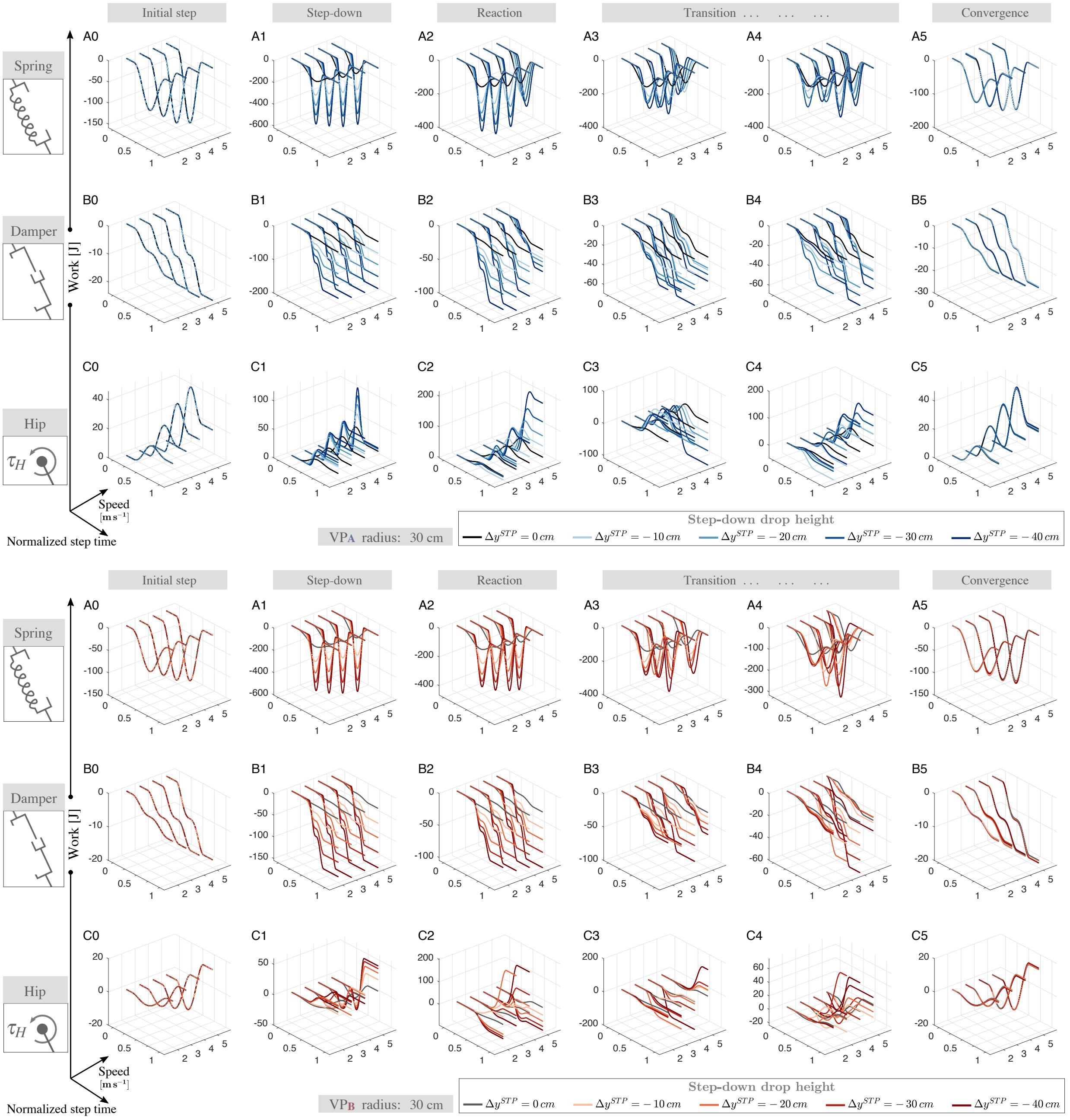

Figure A1: The work performed by the leg spring (**A**), leg damper (**B**) and the hip actuator (**C**) for 30 cm step-down perturbation. The VP controller target is above ($\mathrm{VP_A}$) and below ($\mathrm{VP_B}$) the CoM, respectively.

A3. Downhill Terrain

The time progression of the work performed by the hip and leg for downhill terrain conditions are presented in Figure A2.

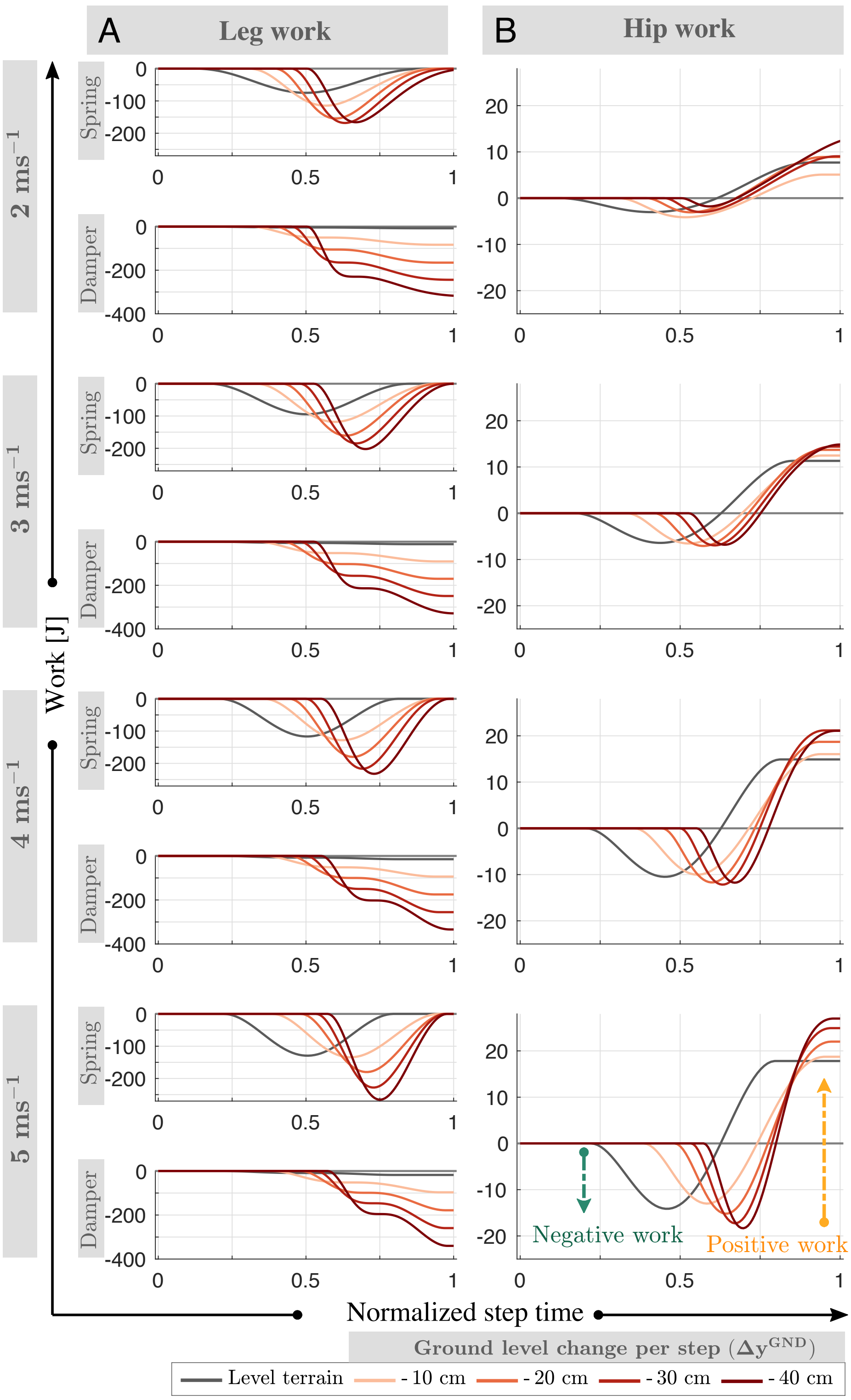

Figure A2: The work performed by the leg (**A**) and hip (**B**) for downhill running with a $\mathrm{VP_B}$ control target at speeds of 2-5 m s^{-1}. The stored/recoiled leg spring energy and dissipated damping energy increase with terrain grade and speed. The negative hip work and net energy generated by the hip follows a similar trend.

References

de Leva, P. (1996). Adjustments to zatsiorsky-seluyanov's segment inertia parameters. *Journal of Biomechanics* 29, 1223–1230. doi: 10.1016/0021-9290(95)00178-6

Drama, Ö. and Badri-Spröwitz, A. (2019). Trunk pitch oscillations for joint load redistribution in humans and humanoid robots. In *2019 IEEE-RAS 19th International Conference on Humanoid Robots*. 531–536. doi: 10.1109/Humanoids43949.2019.9035042

McMahon, T. A. and Cheng, G. C. (1990). The mechanics of running: How does stiffness couple with speed? *Journal of Biomechanics* 23, 65–78. doi: 10.1016/0021-9290(90)90042-2

Sharbafi, M. A. (2017). *Bioinspired template-based control of legged locomotion*. Ph.D. thesis, Technical University of Darmstadt

Sharbafi, M. A., Maufroy, C., Ahmadabadi, M. N., Yazdanpanah, M. J., and Seyfarth, A. (2013). Robust hopping based on virtual pendulum posture control. *Bioinspiration & Biomimetics* 8. doi: 10.1088/1748-3182/8/3/036002

Wojtusch, J. and von Stryk, O. (2015). Humod - a versatile and open database for the investigation, modeling and simulation of human motion dynamics on actuation level. In *Proceedings of the IEEE-RAS International Conference on Humanoid Robots*. 74–79. doi: 10.1109/HUMANOIDS.2015.7363534
