# Supplementary material for: Virtual Point Control for Step-Down Perturbations and Downhill Slopes in Bipedal Running
Source: Front Bioeng Biotechnol. 2020 Dec 18;8:586534. doi: 10.3389/fbioe.2020.586534 (PMC7775500; doi:10.3389/fbioe.2020.586534)
Supplement: Supplementary file 2 [file Data_Sheet_2.zip › data_info.pdf]

Information for the Data Supplement

Article: Virtual Point Control for Step-down Perturbations and Downhill Slopes in Bipedal Running.
Authors: Özge Drama and Alexander Badri-Spröwitz.
Journal: Frontiers in Bioengineering and Biotechnology.

1. Data Format for Gaits

The raw data is provided in “data.mat”, which outputs a Matlab struct called “data”. Due to restrictions in submission size “data.mat” is split into 3 parts: “data_01_flat_terrain.mat”, “data_02_step_down.mat”, “data_03_downhill.mat”.

Struct Structure: single_gait = data.(terrain_type).(vp_method).(running_speed).(change_in_terrain_height)

- Sub-fields of terrain_type: [flat_terrain, step_down, downhill]
- Sub-fields of vp_method: [VPa, VPb]
- Sub-fields of running_speed: [dx_2_mps, dx_3_mps, dx_4_mps, dx_5_mps]
- Sub-fields of change_in_terrain_height: [Dh_0cm, Dh_10cm, Dh_20cm, Dh_30cm, Dh_40cm]

2. Data for Each “single_gait”

2a. Model parameters

- single_gait.m: body mass [kg]
- single_gait.j: moment of inertia [kgm^2]
- single_gait.l: rest length of the effective leg [m]
- single_gait.k: leg spring stiffness [N/m]
- single_gait.c: leg damping coefficient [Ns/m]

2b. Time vectors

- single_gait.t: time vector [s]
- single_gait.t_APTD: time vector between apex and touch-down events
- single_gait.t_TDTO: time vector between touch-down and take-off events
- single_gait.t_TOAP: time vector between take-off and apex events

2c. Kinematics

- single_gait.q: state vector of the center of mass (CoM) q = [xc[m] yc[m] thc[rad] dxc[m/s] dyc[m/s] dth[rad/sec]]
- single_gait.q_APTD: q between apex and touch-down events
- single_gait.q_TDTO: q between touch-down and take-off events
- single_gait.q_TOAP: q between take-off and apex events
- single_gait.qF: horizontal position of the foot at touch-down
- single_gait.lL: leg length [m]
- single_gait.dlL: leg length velocity [m/s]
- single_gait.thL: leg angle [rad]
- single_gait.sthL: leg angular velocity [rad/s]

2d. Kinetics

- single_gait.GRF: horizontal and vertical ground reaction forces GRF= [GRFx[N] GRFy[N]]
- single_gait.Fsp: leg spring force in cartesian coordinates Fsp = [Fspx[N] Fspy[N]]
- single_gait.Fsp: leg damping force in cartesian coordinates Fdp = [Fdpx[N] Fdpy[N]]
- single_gait.Thp: hip torque [Nm]

Example: In Matlab, typing “data.step_down.VPa.dx_3_mps.Dh_20cm.q” would give the CoM state vector for running over a 20 cm step-down drop with 3m/s speed using VPa control approach.
